# Supplementary material for: Feedback control of the heat shock response by spatiotemporal regulation of Hsp70
Source: bioRxiv. 2024 Jan 9:2024.01.09.574867. Preprint. [Version 1] doi: 10.1101/2024.01.09.574867 (PMC10802473; doi:10.1101/2024.01.09.574867)

bioRxiv preprint doi: https://doi.org/10.1101/2024.01.09.574867; this version posted January 9, 2024. The copyright holder for this preprint (which was not certified by peer review) is the author/funder, who has granted bioRxiv a license to display the preprint in perpetuity. It is made available under aCC-BY 4.0 International license.

Figure S1. HS-induced expression dynamics of the Hsf1 target genes

A) Scatter plot of the expression of the HSR genes as measured by RNA sequencing versus mScarlet expression.

B) Relative expression dynamics for Hsf1 target genes, captured with mscarlet fluorescent reporter, over a heat shock time course. Hsf1 target genes are grouped by putative gene function. Each data point and error bar represents the mean and standard deviation of 3 biological replicates.

Figure S2. Hsf1 activity dynamics after HSE deletion

A) Heat shock time courses for the eight ΔHSE mutants with four-hour HSE-YFP fold change outside of the statistically significant WT range. Each data point represents the mean and standard deviation of three biological replicates. Dotted line represents the average of 45 WT biological replicates, the gray shaded area represents the standard deviation of those replicates.

B) Relative non-stress levels of HSE-YFP reporter (normalized to WT) in each ΔHSE induction mutant. Each bar height represents the mean of three biological replicates, error bars represent the SD.

Figure S3. Validating Ydj1 and Apj1 induction mutants

A) Relative basal HSE-YFP (normalized to basal HSE-YFP in WT) when Ydj1 is expressed under non-inducible promoters of various strengths.

B) Relative basal Ydj1 expression measured by mScarlet fluorescence.

C) Basal Apj1 expression measured by mScarlet fluorescence in $apj1_{\Delta HSE}$ vs WT. Statistics: $p < 0.01$. Bar height represents the mean of 3 biological replicates, error bars represent the standard deviation.

bioRxiv preprint doi: https://doi.org/10.1101/2024.01.09.574867; this version posted January 9, 2024. The copyright holder for this preprint (which was not certified by peer review) is the author/funder, who has granted bioRxiv a license to display the preprint in perpetuity. It is made available under aCC-BY 4.0 International license.

D) Basal Hsf1 activity measured by HSE-YFP fluorescent reporter in Apj1ΔHSE vs WT. Bar height represents the mean of 3 biological replicates, error bars represent the standard deviation. Statistics: ns is defined as $p > 0.05$.

Figure S4. Residuals of model fits

The residual values (quantity left unfit by the model) as a function of the parameter sweeps for each ΔHSE mutant and wild type.

Figure S5. Growth curves of cells in ethanol

A) Cells were grown at the indicated concentrations.

B) Max growth rates were fit to a Hill function.

bioRxiv preprint doi: https://doi.org/10.1101/2024.01.09.574867; this version posted January 9, 2024. The copyright holder for this preprint (which was not certified by peer review) is the author/funder, who has granted bioRxiv a license to display the preprint in perpetuity. It is made available under aCC-BY 4.0 International license.

FIGURE S1

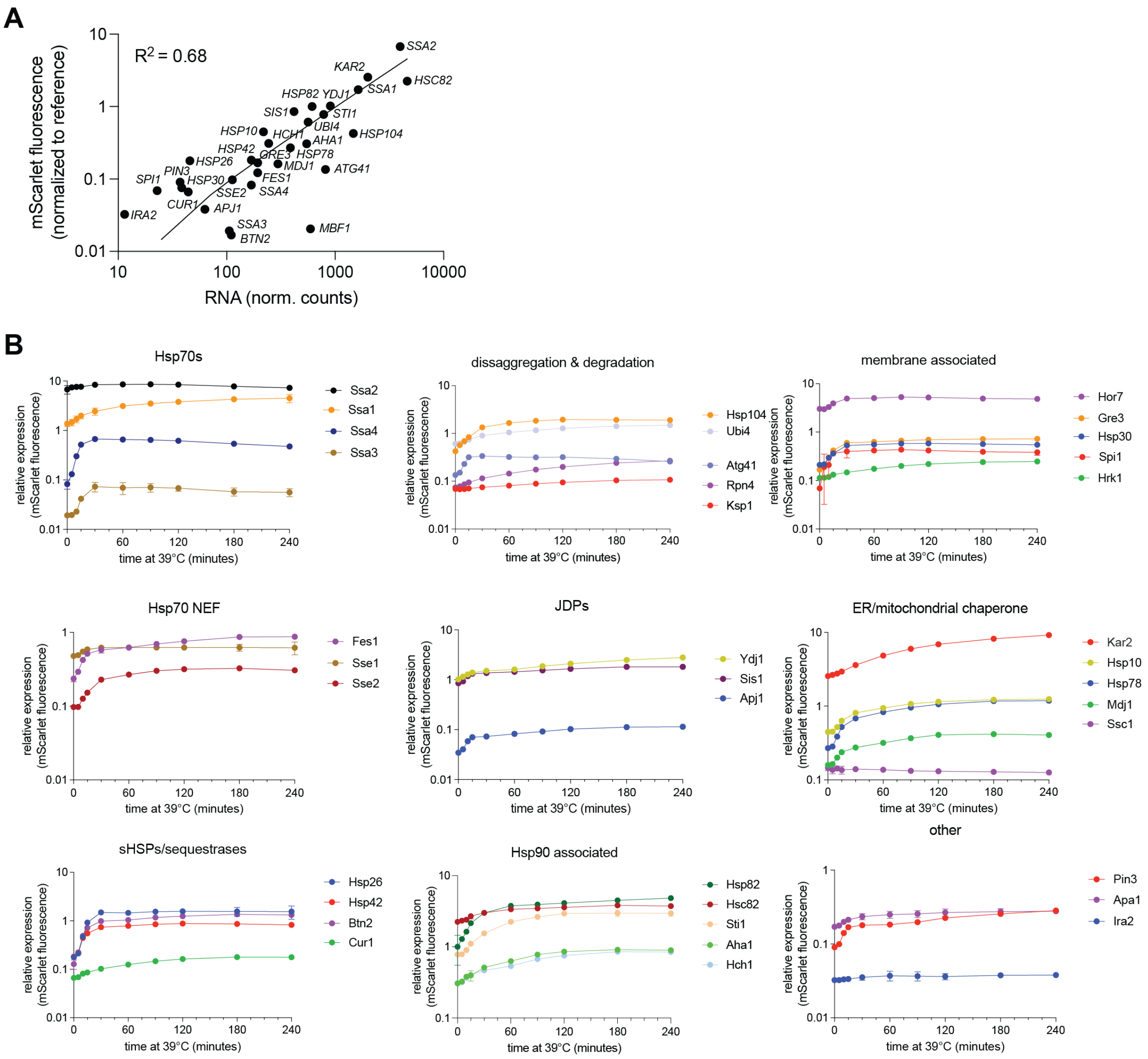

bioRxiv preprint doi: https://doi.org/10.1101/2024.01.09.574867; this version posted January 9, 2024. The copyright holder for this preprint (which was not certified by peer review) is the author/funder, who has granted bioRxiv a license to display the preprint in perpetuity. It is made available under aCC-BY 4.0 International license.

FIGURE S2

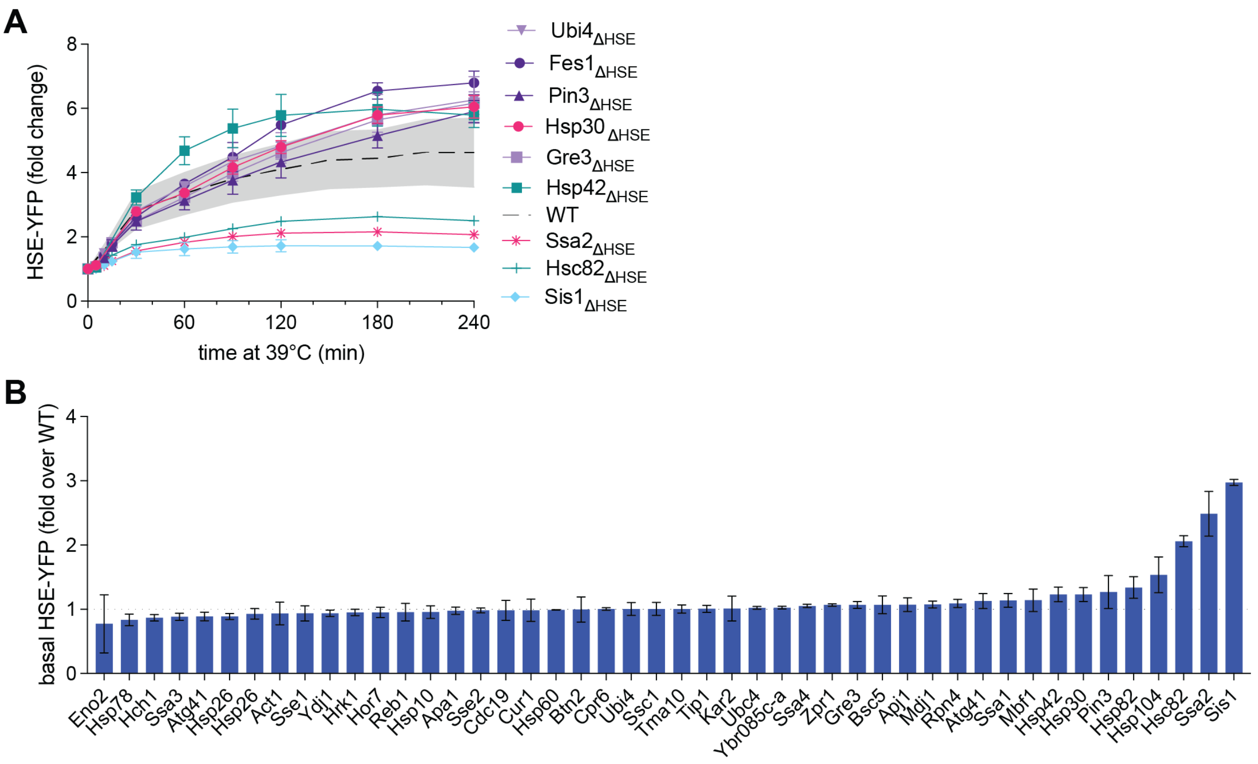

FIGURE S3

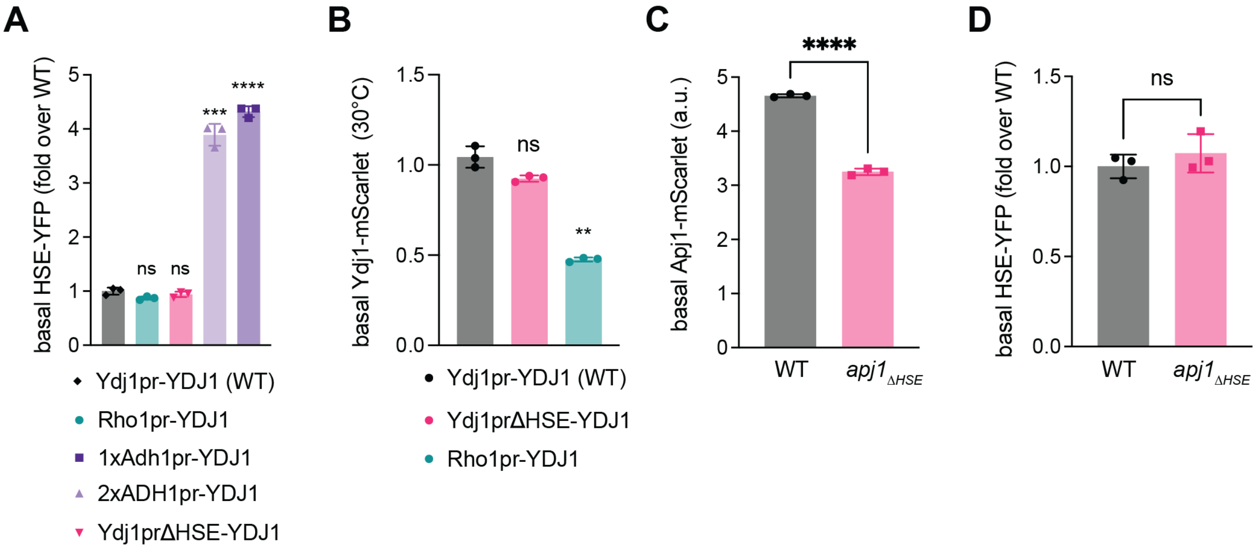

bioRxiv preprint doi: https://doi.org/10.1101/2024.01.09.574867; this version posted January 9, 2024. The copyright holder for this preprint (which was not certified by peer review) is the author/funder, who has granted bioRxiv a license to display the preprint in perpetuity. It is made available under aCC-BY 4.0 International license.

FIGURE S4

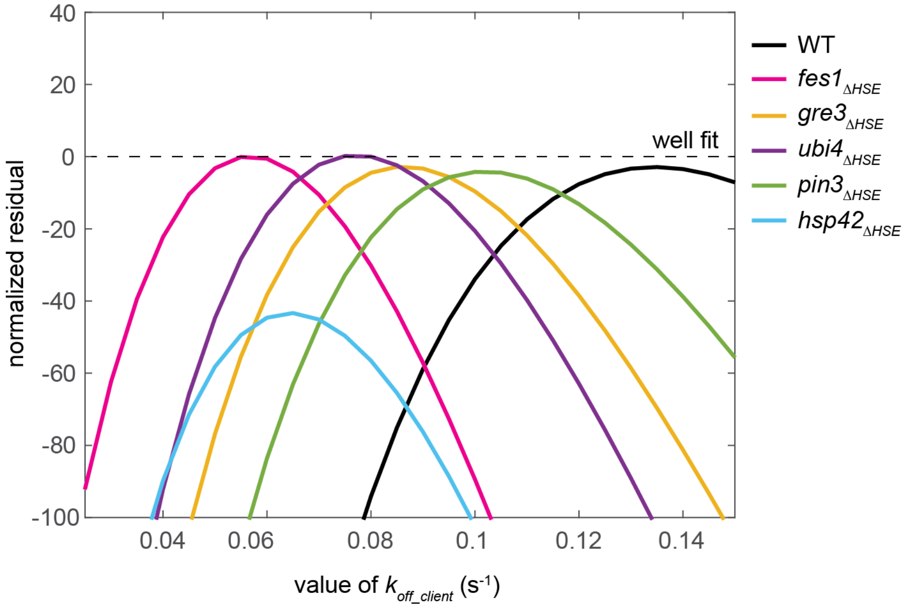

FIGURE S5

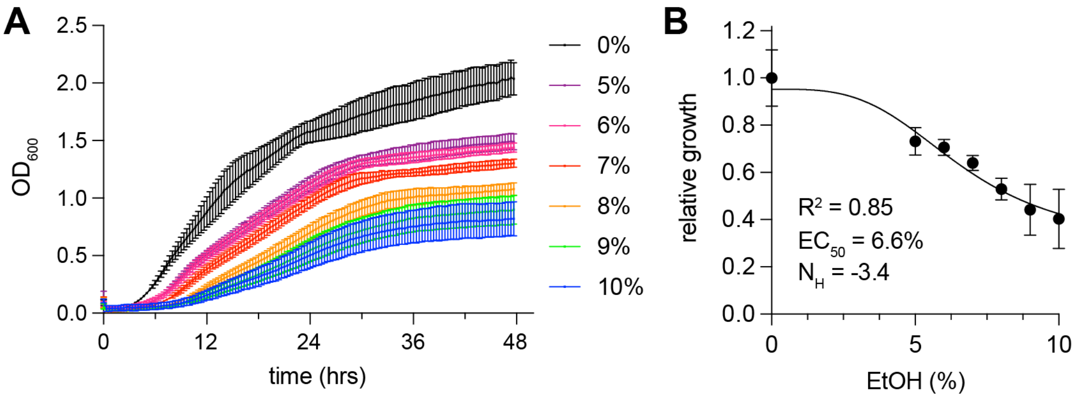

Supplement: 1 [file NIHPP2024.01.09.574867v1-supplement-1.pdf]
